# Post-mortem validation of *in vivo* TSPO PET as a microglial biomarker

**DOI:** 10.1093/brain/awaf078

**Published:** 2025-02-26

**Authors:** Sasvi S Wijesinghe, James B Rowe, Hannah D Mason, Kieren S J Allinson, Reuben Thomas, Davi S Vontobel, Tim D Fryer, Young T Hong, Mehtap Bacioglu, Maria Grazia Spillantini, Jelle van den Ameele, John T O’Brien, Sanne Kaalund, Maura Malpetti, Annelies Quaegebeur

**Affiliations:** Department of Clinical Neurosciences, University of Cambridge, Cambridge CB2 0PY, UK; Department of Clinical Neurosciences, University of Cambridge, Cambridge CB2 0PY, UK; Medical Research Council Cognition and Brain Sciences Unit, Cambridge CB2 7EF, UK; Cambridge University Hospitals NHS Trust, Cambridge CB2 0QQ, UK; Department of Clinical Neurosciences, University of Cambridge, Cambridge CB2 0PY, UK; Department of Clinical Neurosciences, University of Cambridge, Cambridge CB2 0PY, UK; Cambridge University Hospitals NHS Trust, Cambridge CB2 0QQ, UK; Department of Clinical Neurosciences, University of Cambridge, Cambridge CB2 0PY, UK; Department of Clinical Neurosciences, University of Cambridge, Cambridge CB2 0PY, UK; UK Dementia Research Institute at University of Cambridge, Cambridge CB2 0AH, UK; Department of Clinical Neurosciences, University of Cambridge, Cambridge CB2 0PY, UK; Wolfson Brain Imaging Centre, University of Cambridge, Cambridge CB2 0QQ, UK; Department of Clinical Neurosciences, University of Cambridge, Cambridge CB2 0PY, UK; Wolfson Brain Imaging Centre, University of Cambridge, Cambridge CB2 0QQ, UK; Department of Clinical Neurosciences, University of Cambridge, Cambridge CB2 0PY, UK; Department of Clinical Neurosciences, University of Cambridge, Cambridge CB2 0PY, UK; Department of Clinical Neurosciences, University of Cambridge, Cambridge CB2 0PY, UK; Medical Research Council Mitochondrial Biology Unit, Cambridge CB2 0XY, UK; Department of Psychiatry, University of Cambridge, Cambridge CB2 8AH, UK; Centre for Neuroscience and Stereology, Bispebjerg University Hospital, Copenhagen, DK-2400, Denmark; Centre for Neuroscience and Stereology, Copenhagen University Hospital, Copenhagen, DK-2100, Denmark; Department of Clinical Neurosciences, University of Cambridge, Cambridge CB2 0PY, UK; Cambridge University Hospitals NHS Trust, Cambridge CB2 0QQ, UK; UK Dementia Research Institute at University of Cambridge, Cambridge CB2 0AH, UK; Department of Clinical Neurosciences, University of Cambridge, Cambridge CB2 0PY, UK; Cambridge University Hospitals NHS Trust, Cambridge CB2 0QQ, UK

**Keywords:** neuroinflammation, tauopathies, TSPO, PET, microglia, post-mortem

## Abstract

Neuroinflammation is a feature of many neurodegenerative diseases and is quantified *in vivo* by PET imaging with radioligands for the translocator protein (TSPO, e.g. ^11^C-PK11195). TSPO radioligand binding correlates with clinical severity and predicts clinical progression. However, the cellular substrate of altered TSPO binding is controversial and requires neuropathological validation.

We used progressive supranuclear palsy (PSP) as a demonstrator condition, to test the hypothesis that ^11^C-PK11195 PET reflects microglial changes. We included people with PSP-Richardson's syndrome who had undergone ^11^C-PK11195 PET in life (*n* = 8). In post-mortem brain tissue from the same participants, we characterized cell-type specific TSPO expression and quantified microgliosis in eight cortical and 11 subcortical regions.

Double-immunofluorescence labelling for TSPO and cell markers showed TSPO expression in microglia, astrocytes and endothelial cells. Microglial (and not astrocytic) TSPO levels were higher in donors with PSP compared to control subjects (*n* = 3), and correlated with changes in microglial burden. There was a significant positive correlation between regional ^11^C-PK11195 binding potential ante-mortem and the burden of post-mortem CD68+ phagocytic microglia, as well as microglial TSPO levels.

We conclude that *in vivo* disease-related changes in ^11^C-PK11195 binding is largely driven by microglia and can be interpreted as a biomarker of microglia-mediated neuroinflammation in tauopathies.

## Teaser

TSPO PET has been used to measure inflammation in dementia, but the cellular substrate of altered TSPO binding is unclear. Wijesinghe *et al.* used PET imaging and post-mortem brain tissue from individuals with progressive supranuclear palsy to show that microglia are the key immune cells driving TSPO PET signal changes.

## Introduction

Neuroinflammation has emerged as an important pathological feature of multiple neurodegenerative diseases, alongside accumulation of misfolded protein aggregates, synapse loss and neuronal death.^1^ Microglia have gained particular interest as a dominant part of the central neuroinflammatory response.^2^ In tauopathies such as progressive supranuclear palsy (PSP), post-mortem evidence has associated microglial burden to neurodegeneration and tau pathology.^3^ In tau models, microglia respond to tau by releasing inflammatory mediators and attempting to clear aggregates, while prolonged exposure can lead to a dysregulated response, exacerbating tau spread.^4^ Given the potential for prognostication and therapeutic targeting of inflammation, biomarkers that can quantify and localize the inflammatory response *in vivo* are of high importance.^5^

Several measures of neuroinflammation have emerged using PET imaging, with radioligands that bind to the 18 kDa translocator protein (TSPO), such as ^11^C-PK11195.^5^ TSPO PET has been used to quantify and localize neuroinflammatory changes in the brain of people with neurodegenerative diseases, with increased TSPO radioligand binding reported across multiple diseases, including PSP.^5-11^ The regional distribution of increased TSPO radioligand binding is disease-specific (e.g. high in pallidum, midbrain and frontal cortex in PSP^10^), relates to clinical severity, and predicts clinical progression in patients with PSP Richardson's syndrome,^9^ frontotemporal dementia^12^ and amnestic Alzheimer's disease (AD).^13^ Independent studies using different TSPO PET tracers in PSP patients have found similar patterns of increased TSPO signal, inflammation progression and association with different markers of pathology.^6-10,14^

There remain controversies in the interpretation of TSPO PET.^15^ The rationale of TSPO radioligands as biomarkers for neuroinflammation is based largely on autoradiography, animal model and cell culture studies. Early studies correlated TSPO autoradiography to microglial staining in neurotoxin-treated tissue and preclinical mouse models of AD,^16,17^ which was later corroborated by antibody-based primate and mouse studies.^18,19^ However, it has been challenged whether TSPO reflects microglia specifically,^15^ and whether changes in density or reactivity are responsible for this.^20^ Microglia do not consistently upregulate TSPO expression in response to inflammatory stimuli.^20,21^ Moreover, in multiple sclerosis, increased TSPO expression has been observed in endothelium and astrocytes.^22^ This prompts the question as to which cell types contribute to the disease-driven changes observed by TSPO PET imaging.

This study had two principal aims: (i) to test the hypothesis that TSPO elevation in tauopathies is microglial-specific, rather than driven by astrocytes; and (ii) to test the hypothesis that ante-mortem TSPO PET imaging correlates with regional and individual post-mortem differences in microglia. We used PSP as a demonstrator tauopathy because of its high clinicopathological correlation, prognostic relevance of increased TSPO radioligand binding, and short disease course with a short timeframe between PET and death. Confirmation of these hypotheses would support the interpretation of TSPO PET (specifically ^11^C-PK11195) as a microglial neuroinflammatory biomarker in primary tauopathies.

## Materials and methods

Eight people with PSP (ante-mortem diagnosis PSP-Richardson's syndrome) who underwent ^11^C-PK11195 PET during life^8,10^ donated their brain to the Cambridge Brain Bank. Neuropathological diagnosis of PSP was confirmed in all cases. A control group consisted of three age-matched neurologically healthy individuals with mild age-related pathology. Demographics are summarized in Table 1.

**Table 1:** Demographics, clinical and pathological data of PSP donors and controls

Case no	Gender	Age at death (years)	Disease duration (years)	Pathology (stage)	PET-to-death time (months)	PSP rating scale at PET
1	Female	71.9	6.2	PSP (5)	30	46
2	Male	71.0	5.4	PSP (5)	28	33
3	Female	76.4	8.8	PSP (2)	49	46
4	Female	73.7	16.3	PSP (4)	6	74
5	Male	77.4	6.3	PSP (4)	23	54
6	Female	55.0	5.5	PSP (3)	34	29
7	Male	74.5	6.0	PSP (3)	18	39
8	Female	69.9	5.3	PSP (5)	24	38
**Controls**			**Cause of death**			
1	Female	72.4	Malignant tumour of the spinal cord			
2	Female	84.2	Metastatic breast cancer			
3	Female	64.9	Idiopathic pulmonary fibrosis			

PSP = progressive supranuclear palsy.

Patients underwent ^11^CPK11195 PET using dynamic imaging for 75 min on a GE Advance or GE Discovery 690 PET/CT. Non-displaceable binding potential (BP_ND_) was calculated in cortical Brodmann areas and subcortical (modified Hammersmith atlas) regions. Supervised cluster analysis was used to determine the reference tissue time-activity curve and BP_ND_ values were calculated with a simplified reference tissue model.

Double-immunofluorescence labelling in formalin-fixed paraffin-embedded post-mortem brain tissue sections visualized TSPO in astrocytes (GFAP), microglia (IBA1) and endothelium (CD31) in posterior frontal lobe tissue (Brodmann area 6, BA6). Leica^©^ SPE confocal microscopy provided high magnification images and z-stacks. DAB-based immunohistochemistry identified CD68 in eight cortical and 11 subcortical areas. Whole-slide images were acquired with an Aperio AT2 whole-slide scanner (Leica) for immunohistochemistry and a Zeiss Axioscan Z1 Slidescanner for immunofluorescence. QuPath quantified CD68+ staining via pixel-classification in grey and white matter. Area fraction and co-localization analysis of IBA1/TSPO and GFAP/TSPO slides were performed using a colour-thresholding pipeline in ImageJ. Area fraction was defined as the percentage of marker-positive stained area relative to the total tissue area analysed. TSPO area fraction per microglia was calculated by exporting individual cells to ImageJ, converting them to RGB files with individual channels, and utilizing the same colour thresholding pipeline.

**Figure 1:**
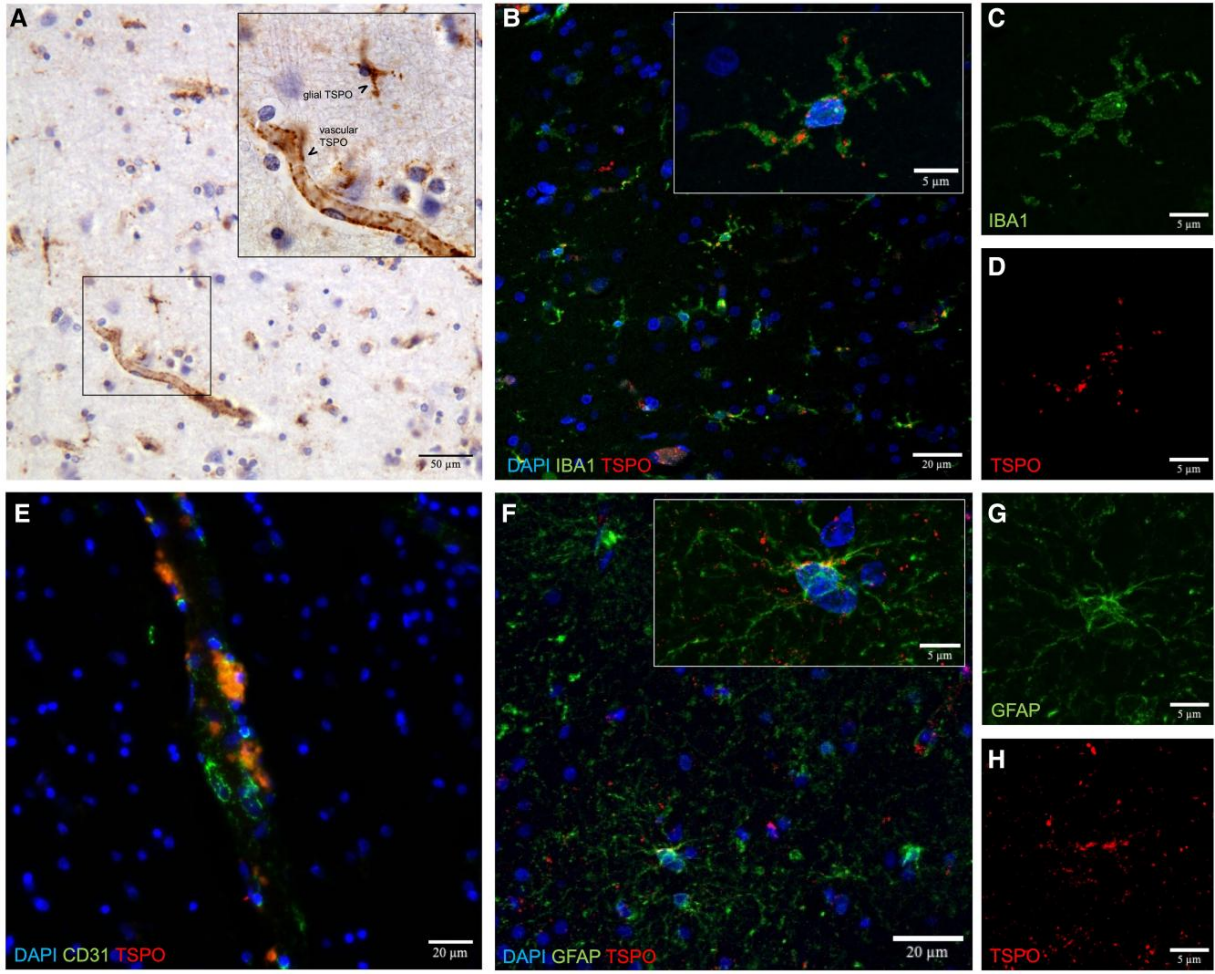
**TSPO multicellular expression in human PSP post-mortem brain.** (**A**) Translocator protein (TSPO) immunohistochemistry in frontal lobe tissue. (**B**) Immunofluorescence images of TSPO (red), microglial marker IBA1 (green) and DAPI (blue). (**C** and **D**) *Insets* of **B**, showing individual channels for (**C**) IBA1 and (**D**) TSPO. (**E**) Immunofluorescence images of TSPO and endothelial marker CD31. (**F**) Immunofluorescence images of TSPO (red), astrocytic marker GFAP (green) and DAPI (blue). (**G** and **H**) *Insets* of **F**, showing individual channels for (**G**) GFAP and (**H**) TSPO. PSP = progressive supranuclear palsy.

Non-parametric testing was applied to account for sample size constraints. Mann–Whitney tests compared control and PSP datasets. The Kruskal–Wallis rank sum and Dunn's *post hoc* test assessed grey/white matter comparisons for IBA1-TSPO and GFAP-TSPO area fractions. A linear mixed-effects model tested the association between *in vivo*  ^11^C-PK11195 binding potential (BP_ND_) and CD68+ microglia quantification across regions. Spearman's correlation analysed the association between (i) microglial TSPO area fraction and total microglia, and (ii) *in vivo*  ^11^C-PK11195 BP_ND_ and TSPO-IBA1 and TSPO-GFAP area fractions in frontal lobe.

Please refer to the online Supplementary material for detailed ‘Materials and methods’.

## Results

### TSPO showed a multicellular expression profile in post-mortem brain tissue

To investigate the cellular substrate of ^11^C-PK11195 binding, we characterized the TSPO expression profile in human post-mortem tissue. Immunohistochemical staining revealed ubiquitous expression of TSPO across white and grey matter of the frontal lobe with a strong expression in the vasculature (Fig. 1A). In addition, non-vascular cellular expression was observed, with staining morphology suggestive of a glial origin.

**Figure 2:**
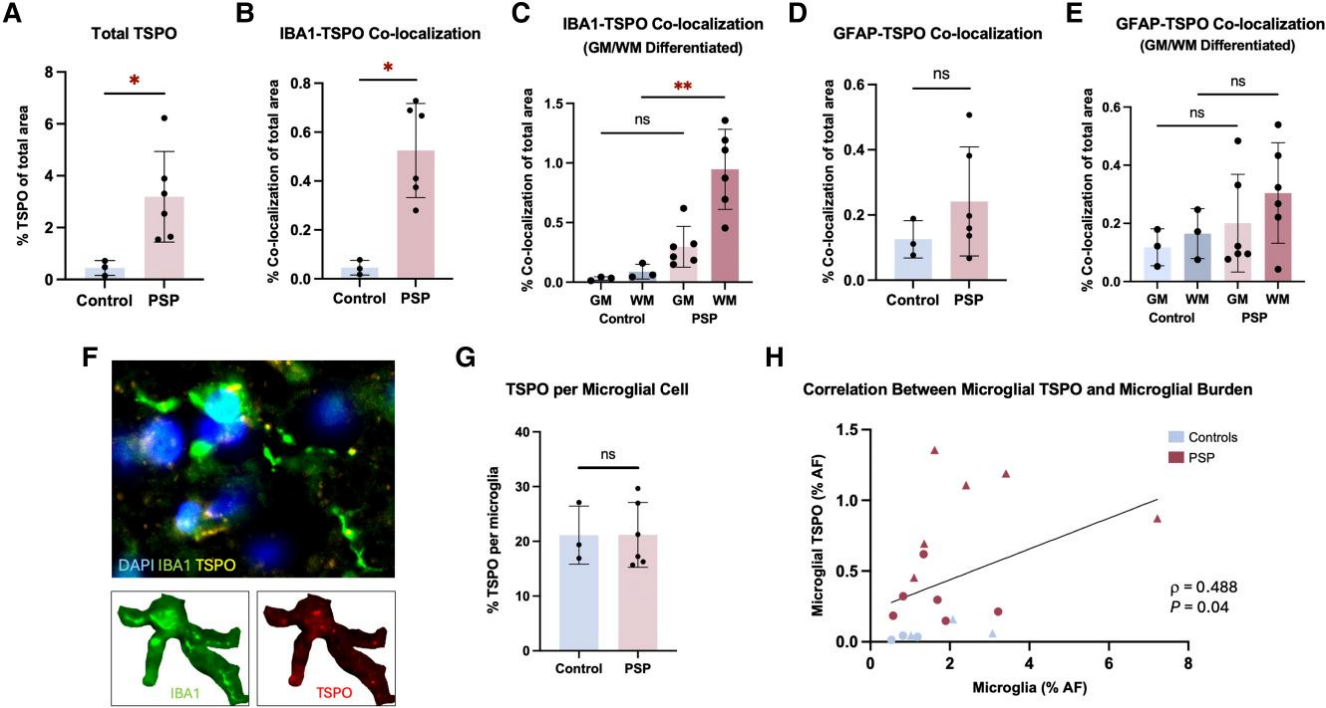
**TSPO expression in people with progressive supranuclear palsy versus controls.** (**A**) Total translocator protein (TSPO) area fraction. (**B** and **D**) Area fraction of TSPO co-localized with (**B**) IBA1 (microglia) or (**D**) GFAP (astrocytes) [Mann–Whitney test; *P* < 0.05; ns = not significant; mean ± standard deviation (SD)]. (**C** and **E**) TSPO co-localization with (**C**) IBA1 or (**E**) GFAP in grey (GM) and white matter (WM) [Kruskal–Wallis test; progressive supranuclear palsy (PSP) GM versus WM, *P* < 0.01]. (**F** and **G**) TSPO per microglia quantification (Mann–Whitney test; *P* < 0.05). (**H**) Microglial TSPO versus total microglia in PSP (red) and controls (blue), grey (circles) and white (triangles) matter (Spearman's correlation; *P* < 0.05).

Cell-type specific expression of TSPO was determined by immunofluorescence labelling for TSPO together with cell-type markers (IBA1 for microglia, GFAP for astrocytes and CD31 for endothelial cells). IBA1-TSPO co-staining revealed TSPO expression in a substantial proportion of the IBA1+ microglia, showing a punctate TSPO pattern in microglial soma and processes across white and grey matter (Fig. 1B–D). CD31-TSPO co-staining showed abundant expression of TSPO in endothelial cells, and this was observed across capillaries, arterioles, venules, arteries and veins (Fig. 1E). GFAP-TSPO co-staining demonstrated sparse astrocytic TSPO expression, with only rare GFAP+ astrocytes showing co-localization with TSPO staining (Fig. 1F–H).

### Quantification of cell-type specific expression revealed a microglia-driven increase of TSPO in PSP

Next, we investigated which cell type drives the increase in TSPO radioligand binding in PSP. As PSP lacks a significant vascular contribution and the cellular substrate of the TSPO signal is mainly contested between the glial cell types,^15^ we focused our analysis on astrocytic and microglial expression. We quantified the area of IBA1-TSPO and GFAP-TSPO co-localization (reflective of microglial and astrocytic TSPO levels, respectively) in posterior frontal lobe (BA6) tissue from neuropathologically confirmed PSP donors who had undergone ^11^CPK11195 PET during life,^8,10^ and control donors.

Quantification of TSPO confirmed an increase in total TSPO levels in PSP brain tissue as compared to controls (Fig. 2A). There was a significant increase in the microglial TSPO levels in PSP versus controls (Fig. 2B). Comparison of microglial TSPO levels in white and grey matter revealed an increase in both compartments in PSP versus controls, although this was more pronounced and statistically significant in white matter (Fig. 2C). Astrocytic TSPO levels did not differ significantly between PSP and control tissue, in frontal lobe as a whole (Fig. 2D) or in white and grey matter compartments (Fig. 2E). These data indicate that the disease-related TSPO increase in PSP is predominantly driven by microglia, rather than astrocytes, in particular microglia in white matter.

To further investigate the basis of increased microglial TSPO levels in PSP tissue, we assessed TSPO area per microglial cell as a measure of cellular expression. There was no difference in the TSPO levels per cell between PSP and controls (Fig. 2F and G). Furthermore, we found a positive correlation between microglial TSPO levels and microglial burden (Fig. 2H), with microglial burden showing a (non-significant) 1.9-fold increase in PSP versus controls (Supplementary Fig. 1). Together, these data (Fig. 2B, G and H) suggest that a higher burden of microglia, rather than TSPO expression per microglial cell, underlies the elevated microglial TSPO levels.

### Post-mortem microgliosis and microglial TSPO levels correlated with ante-mortem TSPO ligand binding

**Figure 3:**
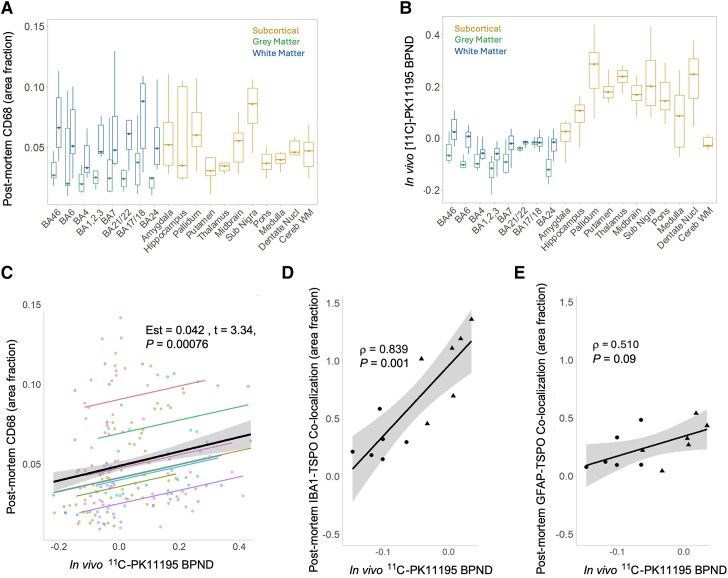
**PET to post-mortem correlation of ^11^C-PK11195 BP_ND_ with CD68+ microglia and TSPO cell-type specific expression.** (**A**) CD68+ microglia area fractions by region in white matter (WM, blue), grey matter (GM, green) and subcortical structures (yellow). (**B**) Regional ^11^C-PK11195 binding potential (BP_ND_) in the corresponding compartments. (**C**) Association between regional CD68 area fractions and ante-mortem ^11^C-PK11195 BP_ND_. Dots represent individual regional values; black line shows the group-level association; coloured lines represent patient-specific associations, estimated by the model. (**D** and **E**) ^11^C-PK11195 BP_ND_ association with (**D**) TSPO-IBA1 and (**E**) TSPO-GFAP co-localization area fractions in posterior frontal cortex (BA6) across grey matter (circles) and white matter (triangles). BA = Brodmann area.

To confirm that TSPO radioligand binding determined with PET in PSP reflects microglial reactivity, we assessed the regional association between histologically determined microgliosis in post-mortem brain tissue and ^11^C-PK11195 binding potential (BP_ND_) from the same PSP donors during life.^8,10^ ‘Phagocytic’ microglia were quantified using CD68 immunohistochemistry across eight cortical and 11 subcortical regions. Area fractions of CD68+ microglia were the highest across the cortical white matter regions and subcortical regions (Fig. 3A). ^11^C-PK11195 BP_ND_ was calculated for each individual donor for each region (Fig. 3B). The model comparison of three linear mixed effects models identified one with a fixed term for the effect of ^11^C-PK11195 BP_ND_ on CD68 area fraction, and random intercept for individual patients [ΔChi-square (1) = 4.47, *P* = 0.0345], but not random slope [ΔChi-square (1) = 1.89, *P* = 0.39]. The optimal model confirmed the significant positive association between *in vivo* TSPO radioligand binding and the area fraction of post-mortem CD68-positive ‘phagocytic’ microglia (Est = 0.042, *t* = 3.43, *P* = 0.00076; Fig. 3C). The association remained significant (Est = 0.042, *t* = 3.43, *P* = 0.00077) with the addition of covariates for the interval from PET scanning to death (Est = 0.0009, *t* = 1.41, *P* = 0.219) and PSP pathology stage (Est = −0.0035, *t* = −0.47, *P* = 0.656).^23^

There was a significant positive association between *in vivo* TSPO radioligand binding and microglial TSPO levels in the frontal lobe (Spearman's ρ = 0.839, *P* = 0.001; Fig. 3D), whilst an association with astrocytic TSPO levels was not significant (Spearman's ρ = 0.510, *P* = 0.09; Fig. 3E).

## Discussion

This is the first study to show TSPO PET-to-pathology correlations using post-mortem brain tissue from participants in ante-mortem ^11^C-PK11195 PET studies. Our key findings are that: (i) despite TSPO's multicellular expression, its increased levels in disease were microglial (over astrocytic) in origin, which correlated with microglial burden; and (ii) TSPO radioligand binding during life was significantly associated with post-mortem CD68+ microglia and microglial (but not astrocytic) TSPO levels. These findings indicate that TSPO PET in a primary tauopathy can be interpreted as a microglial-specific neuroinflammatory biomarker, supporting interpretations of previous PET studies in PSP reporting increased TSPO radioligand binding.^6-10,14^

TSPO expression in multiple brain cell types was confirmed, consistent with prior studies.^22,24,25^ However, our cell-type specific TSPO expression studies identified microglia as the key driver of increased TSPO levels in disease. Our finding that microglial burden (including CD68+) rather than elevated TSPO expression per cell contributes to this increase is consistent with some of the recent findings in the field.^20^ In brain donors who underwent ^11^C-PK11195 PET during life, we compared post-mortem quantifications of microglia and cell-type specific TSPO levels with their corresponding *in vivo* TSPO radioligand binding. The TSPO PET binding *in vivo* correlated with CD68-positive phagocytic microglia post-mortem, and did so across multiple cortical and subcortical brain regions. In the frontal cortex, we confirmed the association of *in vivo* TSPO radioligand binding with microglial TSPO levels post-mortem, while no such association was found with astrocytic TSPO. Taken together, these findings indicate that while TSPO radioligand binding likely reflects contributions from various TSPO-expressing cell types, its increase in PSP is primarily of microglial origin. This supports the interpretation of TSPO PET as a microglia-specific neuroinflammatory biomarker in the primary tauopathy PSP.

We recognize the cellular substrate and mechanism driving increased TSPO radioligand binding might be disease-specific, with reports suggesting varying effects on TSPO expression across different diseases, species and models.^20,22,24-27^ Further work is required to exclude the possibility that the association between microglia and TSPO radioligand binding is PSP/tauopathy-specific, with PET-to-pathology comparisons needed in other neurodegenerative disorders. Few studies have reported TSPO expression in a range of microglial phenotypes^20,22,26^ with a recent study showing an association with ‘phagocytic’ microglia.^27^ Our finding that TSPO PET radioligand binding correlates with CD68+ microglia is aligned with the latter.

Our study has potential limitations, in relation to sample size, diagnostics and ligand specificity. Despite the relatively small sample size, we had adequate power, given (i) the large effect size expected from previous PET-only studies in PSP at the time of death (Cohen's *d* is often >2)^8,10,28^; and (ii) the use of a linear mixed-effect model for PET-to-pathology analyses, leveraging all 19 regional data-points available for the eight PSP donors. In addition, the correlation of TSPO PET to CD68+ microglia in every single case of our series, provides evidence to support the inference of generalization.^29^ Our study lacked power to confirm the mild effects of age and sex on TSPO radioligand binding,^6,30^ but we note that the relationship between the PET and CD68 signals was present in all participants, regardless of age and sex.

PSP-Richardson's syndrome offers advantages as a demonstrator condition due to its strong clinicopathological correlation, relatively short PET-to-brain donation, and absence of a major vascular contributions, unlike AD. We used the first generation TSPO ligand ^11^C-PK11195. While second (e.g. ^11^C-PBR28) and third (e.g. ^18^F-GE180) generation tracers may offer an improved signal-to-noise ratio,^5^ they are confounded by genetic polymorphisms affecting binding affinity. They are therefore less suitable for rare diseases, where gene-stratified recruitment would be especially challenging.

In summary, our findings support the use of TSPO PET as a microglia-specific neuroinflammatory biomarker in the primary tauopathy of PSP. Microglial TSPO levels contribute to *in vivo*  ^11^C-PK11195 binding, over and above astrocytic levels. Given the PET evidence of increased TSPO radioligand binding in core pathological regions, and its predictive value for clinical decline, we suggest that TSPO PET can be used to quantify microglial-mediated neuroinflammation in primary tauopathies such as PSP, assisting the design of clinical trials with disease-modifying therapies.

## Supplementary Material

awaf078_Supplementary_Data

## Data Availability

Anonymized post-mortem and PET data used for this analysis are available on request. Further participant-specific information, images or samples can be requested but are likely to require a data/material transfer agreement to adhere to consent restrictions including protection of confidentiality. For the purpose of open access, the authors have applied a Creative Commons Attribution (CC BY) license to any Author Accepted Manuscript version arising from this submission.
