# Supplementary material for: Post-mortem validation of in vivo TSPO PET as a microglial biomarker
Source: Brain. 2025 Feb 26;148(6):1904–10. doi: 10.1093/brain/awaf078 (PMC12129730; doi:10.1093/brain/awaf078)
Supplement: awaf078_Supplementary_Data [file awaf078_supplementary_data.zip › brain-2024-01269-File006.pdf]

Supplementary Material 1

Detailed Materials and Methods

Human brain samples and donors

Eight PSP subjects (with *ante-mortem* diagnosis of PSP-Richardson's syndrome) who underwent [^{11}C]-PK11195 PET during life[1,2] donated their brain to the Cambridge Brain Bank. Neuropathological diagnosis of PSP was confirmed in all cases, as per Rainwater criteria[3], and tau pathology was staged according to Kovacs *et al*[4]. The demographics of the PSP brain donors are summarised in Table 1. Formalin-fixed paraffin-embedded tissue sections from eight cortical and eleven subcortical areas (Brodmann areas BA46, BA6, BA4, BA1,2,3, BA7, BA21/22, BA17/18, BA24, hippocampus, amygdala, putamen, pallidum, thalamus, midbrain, substantia nigra, pons, medulla, dentate nucleus, cerebellar white matter) of eight PSP donors alongside three control cases were provided by the Cambridge Brain Bank (Neuropathology Research in Dementia protocol, Research Ethics Committee reference 16/WA/0240). The control group consisted of age-matched neurologically healthy controls with minimal to mild age-related pathology.

In vivo neuroimaging data

Full details of the imaging protocol and data acquisition are published elsewhere[1,2]. Briefly, patients underwent [^{11}C]-PK11195 PET using dynamic imaging for 75 minutes on a GE Advance or GE Discovery 690 PET/CT (GE Healthcare, Waukesha, USA), together with volumetric 3T magnetic resonance imaging (MRI) on a Siemens Magnetom Tim Trio or Verio (Siemens Healthineers, Erlangen, Germany). For each participant, the aligned dynamic PET image series for each scan was rigidly co-registered to the T1-weighted MRI image. Non-displaceable binding potential (BP_{ND}) was calculated in cortical and subcortical regions of interest using two atlases: (1) Brodmann areas (www.nitrc.org/projects/mricron) for cortical regions; and (2) a modified version of the n30r83 Hammersmith atlas (www.brain-development.org), which includes brainstem parcellation and the cerebellar dentate nucleus. Supervised cluster analysis was used to determine the reference tissue time-activity curve and BP_{ND} values were calculated in each ROI using a simplified reference tissue model with vascular binding correction.

Histological staining

We used DAB-based immunohistochemical staining to visualise expression of TSPO and CD68. CD68 was prioritized as a marker for the PET-to-postmortem correlation analysis because of its relationship with 'activated', phagocytic microglia[5,6] and disease-related change in microglia[7]**.** Slides were deparaffinized, washed and underwent antigen retrieval at 60°C for 20 minutes with sodium citrate (pH 6.2) for the CD68 and TRIS-EDTA (pH 9.2) for the TSPO protocol. Slides were quenched with 3% hydrogen peroxide and blocked with 5% Normal Horse Serum. Slides were incubated overnight with primary antibodies (TSPO: EPR5384, Abcam, 1:500; CD68: M0876, DAKO, 1:50). Following incubation with secondary antibodies (TSPO: ImmPRESS® HRP Horse Anti-Rabbit IgG Polymer Detection Kit, Peroxidase, Vector Laboratories, MP-7401; CD68: Leica BOND Polymer Refine Detection Kit, DS9800). ABC solution and DAB were used (VECTASTAIN® ABC-AP Kit, Vector Laboratories, AK-5000; DAB Substrate Kit, Vector Laboratories, SK-4100). Slides were counterstained with haematoxylin and then dehydrated and mounted using DPX. CD68 was quantified across all 8 cortical and 11 subcortical regions in grey and white matter separately.

Cell-type specific expression studies were carried out by immunofluorescence co-staining of TSPO with pan-cell-type markers (IBA1 for microglia, GFAP for astrocytes and CD31 for endothelial cells). Slides from the BA6 area were baked for two hours and then soaked in xylene, 100%, 96%, 70% and 50% ethanol in series. Antigen retrieval with TRIS-EDTA (pH 9.2) was done at 90°C for 20 minutes, after which blocking with 2.5% BSA for 1 hour was followed by overnight incubation with primary antibodies (IBA1: A82670, antibodies.com, 1:200; GFAP: A83720, antibodies.com, 1:200; TSPO: EPR5384, Abcam, 1:500; CD31: AB9498, Abcam, 1:500). Biotum TrueBlack® Lipofuscin Autofluorescence Quencher (A11055, Invitrogen) was used to reduce autofluorescence and secondaries were added (A11055, Invitrogen: 1:250 for IBA1, 1:500 for GFAP, 1:250 for CD31; A10042, Invitrogen: 1:250 for TSPO). The slides were stained with DAPI and mounted using Fluoromount.

Imaging and quantification

A Leica© SPE Confocal Microscope was used for high magnification images and z-stacks. Whole-slide images were acquired by an Aperio AT2 whole slide scanner (Leica) at x40 magnification for immunohistochemistry slides, and a Zeiss Axioscan Z1 Slidescanner at x40 for immunofluorescence slides.

Area fraction and co-localisation analysis of the IBA1/TSPO and GFAP/TSPO whole slide-scans was performed using a colour-thresholding pipeline in ImageJ. 16-bit images of the white and grey matter ROIs excluding DAPI were used to derive respective RGB colour files, used to quantify co-localisation and total area. Thresholds for each parameter were optimised to the grey and white matter ROIs. Co-localisation colour thresholding was specifically determined as yellow signal i.e., overlapping red (TSPO) and green (GFAP/IBA1) signals. TSPO per microglia analysis was done in a similar manner, where individual cells were selected at full resolution in QuPath and exported to ImageJ (20 cells per case were evaluated). Then, colour thresholding was utilised to select for the red (TSPO) and green (microglia) signals for the % ratio to be calculated per cell, then an average determined for each case. Case 2 and Case 6 did not undergo IBA1-TSPO and GFAP-TSPO analysis respectively, as staining result was unsatisfactory. Case 8 only had sections available for post-mortem CD68 staining.

QuPath was used to quantify CD68+ staining through pixel-classification based analysis in grey and white matter differentiated ROIs. Thresholding for each antibody was then applied to individual images with a gaussian pre-filter, smoothing sigma of 0.5, and a greyscale intensity less than 80. The area fraction was determined as percentage of stained area relative to total tissue area.

Statistical Analysis

Mann-Whitney tests were conducted on the relevant comparisons between control and PSP datasets. The Kruskal-Wallis rank sum test and Dunn's post-hoc test were applied to the grey matter/white matter comparisons for IBA1-TSPO and GFAP-TSPO area fraction analysis.

A linear mixed effects model was performed to investigate the association between *in vivo* [^{11}C]-PK11195 BP_{ND} and CD68+ microglia quantification across all regions. Specifically, CD68 quantification was included as dependent variable and [^{11}C]-PK11195 BP_{ND} as fixed factor, while random effects accounted for patient individual variability. As for our previous work[1], using analysis-of-deviance, we compared three models using analysis-of-deviance: (1) an initial model with only a random intercept term for patients, (2) a model also including the fixed effect of regional [^{11}C]-PK11195 BP_{ND} (x variable) on CD68 quantification (y variable), and (3) a model with both random intercept and random slope terms. We used the *anova()* R function to compare the fitted models, based on changes in Chi-square and the Akaike information criterion. Spearman's correlation analyses were performed to assess the

association between the *in vivo* [^{11}C]-PK11195 BP_{ND} and TSPO-IBA1 co-localisation and TSPO-GFAP co-localisation area in frontal lobe as well as the association between the microglial TSPO expression and total microglial area.

References

1. Malpetti M, Passamonti L, Rittman T, et al. Neuroinflammation and Tau Colocalize in vivo in Progressive Supranuclear Palsy. *Ann Neurol*. 2020;88(6):1194-1204. doi:10.1002/ana.25911

2. Passamonti L, Rodríguez PV, Hong YT, et al. [11C]PK11195 binding in Alzheimer disease and progressive supranuclear palsy. *Neurology*. 2018;90(22):e1989-e1996. doi:10.1212/WNL.0000000000005610

3. Roemer SF, Grinberg LT, Crary JF, et al. Rainwater Charitable Foundation criteria for the neuropathologic diagnosis of progressive supranuclear palsy. *Acta Neuropathol (Berl)*. 2022;144(4):603-614. doi:10.1007/s00401-022-02479-4

4. Kovacs GG, Lukic MJ, Irwin DJ, et al. Distribution patterns of tau pathology in progressive supranuclear palsy. *Acta Neuropathol (Berl)*. 2020;140(2):99-119. doi:10.1007/s00401-020-02158-2

5. Zotova E, Bharambe V, Cheaveau M, et al. Inflammatory components in human Alzheimer's disease and after active amyloid-β42 immunization. *Brain J Neurol*. 2013;136(Pt 9):2677-2696. doi:10.1093/brain/awt210

6. Walker DG, Lue LF. Immune phenotypes of microglia in human neurodegenerative disease: challenges to detecting microglial polarization in human brains. *Alzheimers Res Ther*. 2015;7(1):56. doi:10.1186/s13195-015-0139-9

7. Hopperton KE, Mohammad D, Trépanier MO, Giuliano V, Bazinet RP. Markers of microglia in post-mortem brain samples from patients with Alzheimer's disease: a systematic review. *Mol Psychiatry*. 2018;23(2):177-198. doi:10.1038/mp.2017.246
