# Supplementary material for: Post-mortem validation of in vivo TSPO PET as a microglial biomarker
Source: Brain. 2025 Feb 26;148(6):1904–10. doi: 10.1093/brain/awaf078 (PMC12129730; doi:10.1093/brain/awaf078)
Supplement: awaf078_Supplementary_Data [file awaf078_supplementary_data.zip › SupplementaryMaterial2finalproofs.pdf]

Supplementary Material 2

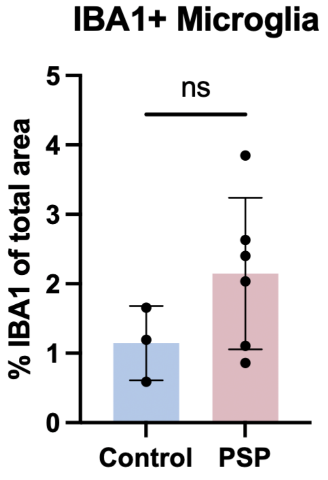

Supplementary Figure 1: Area fraction of IBA1+ microglia in PSP brain tissue versus controls (Mann-Whitney Test; $p = 0.26$; ns = not significant; mean ± SD).
